# Bioactive Compounds Isolated from Marine Bacterium *Vibrio neocaledonicus* and Their Enzyme Inhibitory Activities

**DOI:** 10.3390/md17070401

**Published:** 2019-07-08

**Authors:** Isabel Gómez-Betancur, Jianping Zhao, Lin Tan, Chang Chen, Ge Yu, Paola Rey-Suárez, Lina Preciado

**Affiliations:** 1 Haikou Experimental Station, Chinese Academy of Tropical Agricultural Sciences (CATAS), Haikou 571101, China; 2 Programa Ofidismo-Escorpionismo, Facultad de Ciencias Farmacéuticas y Alimentarias, Universidad de Antioquia, Medellín 1226, Colombia; 3 National Center for National Products Research, School of Pharmacy, University of Mississippi, MS 38677, USA; 4 Key Laboratory of Tropical Marine Bio-resources and Ecology, South China Sea Institute of Oceanology, Chinese Academy of Sciences, Guangzhou 510301, China

**Keywords:** *Vibrio neocaledonicus*, acetylcholinesterase (AChE), alphaglucosidase (AG), xanthine oxidase (XO), indole, bioactive compounds, snake venom

## Abstract

Marine organisms are recognized as a source of compounds with interesting biological activities. *Vibrio neocaledonicus* has been reported on for its high effectiveness against corrosion in metals but it has been little studied for its chemical and biological activities. In this study, four compounds were isolated from *V. neocaledonicus*: indole (**1**); 1H-indole-3-carboxaldehyde (**2**); 4-hydroxybenzaldehyde (**3**) and Cyclo (-Pro-Tyr) (**4**); using a bioassay-guided method, since in a previous study it was found that the ethyl acetate extract was active on the enzymes acetylcholinesterase (AChE), alpha-glucosidase (AG) and xanthine oxidase (XO). The inhibitory activities of the three compounds against AChE, AG and XO was also evaluated. In addition, the enzymatic inhibitory activity of indole to the toxins from the venom of *Bothrops asper* was tested. Results showed that indole exhibited strong inhibitory activity to AG (IC_50_ = 18.65 ± 1.1 μM), to AChE, and XO (51.3% and 44.3% at 50 μg/mL, respectively). 1H-indole-3-carboxaldehyde displayed strong activity to XO (IC_50_ = 13.36 ± 0.39 μM). 4-hydroxybenzaldehyde showed moderate activity to XO (50.75% at 50 μg/mL) and weak activity to AChE (25.7% at 50 μg/mL). Furthermore, indole showed a significant in vitro inhibition to the coagulant effect induced by 1.0 μg of venom. The findings were supported by molecular docking. This is the first comprehensive report on the chemistry of *V. neocaledonicus* and the bioactivity of its metabolites.

## 1. Introduction

Humans are affected by multiple diseases to which researchers are seeking effective and adequate cures. Some of these diseases such as Alzheimer’s disease, diabetes and gout (caused by hyperuricemia) are chronic diseases, which could be harmful to health and even life threatening. The synthesized medicines currently used to treat the aforementioned diseases have some side effects and unwanted adverse reactions, which directly affect the health of patients. Consequently, it is important and necessary to find the active compounds in nature as drug leads. It is known that oceans, with high concentrations of salt and low temperatures, provide an extreme environment for the microorganisms living in them, which force them to develop the capacity to produce special secondary metabolites that help their adaptation. Abundant evidence has shown that marine bacteria in almost all parts of the marine world, whether associated with other organisms or free-living, have developed different strategies to survive in extreme environments [1]. It has been reported that coral-associated bacteria have antimicrobial properties and act as antagonists against pathogens [2,3].

Bacteria belonging to the genus *Vibrio* consist of facultative aerobic and anaerobic gram-negative species, which have a curved morphology, and are generally halodependent. *V. neocaledonicus*, has been reported on for its corrosion inhibiting effect and this result is comparable with some industrial coatings, since this bacterium affects corrosion by the formation of an inhibitory layer on the metal surface [4,5]. Even Vibrios bacteria are dominant in the marine environment with genomic flexibility, and they have not been explored extensively. Some studies have reported that Vibrio bacteria could be the producers of biologically active compounds with interesting antibacterial, anticancer and antiviral properties [6]. We have conducted investigations in our laboratory on *Vibrio* spp. of the Vibrionaceae family, particularly *V. neocaledonicus*, to explore the inhibitors for the acetylcholinesterase (AChE), alpha-glucosidase (AG), and xanthine oxidase (XO), as well as the inhibitory substances for snake venom [7].

Acetylcholinesterase inhibitors (AChEIs) can reduce the activity of the enzyme AChE that degrades the neurotransmitter acetylcholine (ACh). ACh is essential for processing memory and learning. Both the concentration and function of ACh are found to be decreased in patients with neuro degenerative diseases like Alzheimer‘s disease. AChEIs may alter the cholinergic synapse, which is involved in the etiology of Alzheimer’s disease [8]. A large number of compounds with varied structures from natural sources have been found as potential AChEIs [9,10,11]. Although most of those compounds, isolated from plants, algae, fungi, cyanobacteria, marine phytoplankton and marine sessile organisms like sponges and soft corals, have been found to be important sources [12]. 

The search for new inhibitors of the XO and AG enzymes is urgent, due to their correlations with painful diseases such as gout and diabetes, respectively. Urate is the final product of the reaction catalyzed by XO. High levels of urate in blood cause gout, a painful joint disease. Some prescription drugs like allopurinol can be used for the treatment of gout but with side effects such as allergic reaction, gastrointestinal discomfort, diarrhea and drowsiness [13]. The inhibitors of the enzyme AG are used to treat type 2 diabetes because they can lower blood sugar via decreasing the degradation rate of carbohydrates after ingestion.

Snakebite envenoming kills more than 100,000 people and maims more than 400,000 people every year [14]. Viperid snakes of the *Bothrops* genus inflict the majority of snakebites in Central and South America [15]. The pathophysiological effects induced by snakebite envenomations from *Bothrops* are induced by the biological activities of several enzymes, mainly phospholipases A_2_ (PLA_2_), zinc dependent metalloproteinases (SVMPs) and serine proteinases (SVSPs) [16]. SVMPs induce hemorrhage by the proteolytic degradation of endothelial cell surface proteins and extracellular matrix components of capillaries and venules. PLA_2_ hydrolyze the sn2 ester bond of cell membrane glycerophospholipids, inducing systemic and local myotoxicity, mionecrosis and edema. On the other hand, SVSPs have been classified as activators of the fibrinolytic system, procoagulant, anticoagulant and platelet-aggregating enzymes [17,18]. Therapy for snakebite envenomation is mainly based on the intravenous administration of antivenoms [19,20]. However, it has been demonstrated that antivenoms have a limited efficacy against the local tissue damaging activities of venoms [21,22]. Efforts are made to search for alternative sources of venom inhibitors, either synthetic or natural, that would complement the action of antivenoms, for the inhibition of toxins that induce local tissue damage, such as PLA_2s_ and SVMPs. 

In an attempt to explore the biological and chemical aspects of *V. neocaledonicus*, we analyzed two extracts from the same strain but from two different media (2216E and LB). On the other hand, snake bite envenoming is a serious public health problem in many regions of the world. Therefore, we chose four objectives of our preliminary study in the search for bioactive inhibitory substances: AChE, AG, XO and snake venom toxins.

Herein, we describe the identification of the active compounds from the ethyl acetate extract of *V. neocaledonicus*. The production of compounds from two different media (2216E and LB) were compared in the study, and the inhibitory activities of the compounds against the enzymes AG, AChE and XO, as well as the protective properties against the indirect hemolytic, coagulant and proteolytic effects of *Bothrops asper* venom were evaluated. This is the first comprehensive report of chemical investigation on *V. neocaledonicus* and the enzymatic inhibition properties of the isolated chemicals, excluding two patents applications about indole and indole-3-formaldehyde from *V. neocaledonicus.* [23,24]. 

## 2. Results

### 2.1. Culture Medium and Metabolite Production of Vibrio neocaledonicus

**Figure 1:**
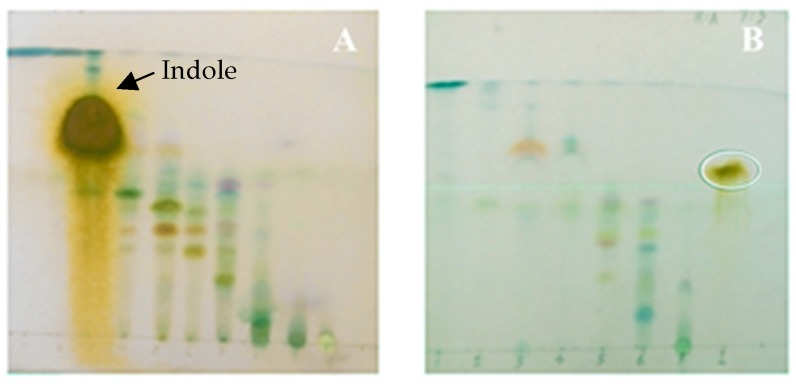
Thin layer chromatography (TLC) of fractions with mobile phase hexane-acetone (7:3) for comparison of the chemical differences between the first (**A**) and the second (**B**) cultures of *V. neocaledonicus*.

In order to compare the effect of different culture media on the production of metabolites of *V. neocaledonius*, we carried out a qualitative thin layer chromatography (TLC) study to analyze the ethyl acetate extract from the strains cultured in 2216E and Lysogeny broth L.B medium (liquid). From Figure 1, the difference of the chemical pattern between fractionation A (extract obtained from medium 2216E) and fractionation B (extract obtained from LB medium) can be observed. The major compound (indole) appeared in fractionation A and it was used as a chemical marker in fractionation B, whereas we did not observe it in the plate corresponding to the second extract (Figure 1B). The plates were revealed with p-anisaldehyde and different colors were observed that correspond mainly to phenolic compounds, terpenes, flavonoids, flavanones. The anisaldehyde with sulfuric acid allowed us to visualize, among other compounds, terpenoids, polypropanoids, saponins and phenolic compounds. In the violet and red colors, terpenoids can be observed, flavonoids in yellow, and phenolic compounds in the brown color (Figure 1A,B).

### 2.2. Structural Elucidation

**Figure 2:**
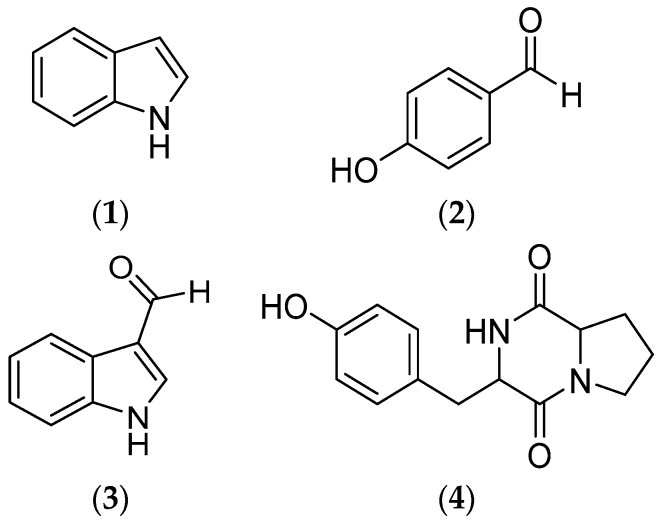
Chemical structures of metabolites isolated from *Vibrio neocaledonicus*.

Chromatographic separation of the ethyl acetate extracts of *V. neocaledonicus* afforded four compounds (Figure 2). Structure elucidation of all compounds was performed by 1D and 2D NMR (results were shown in attachment), and confirmed by comparison with the data from the literature. 

The compounds were identified as indole [25] (**1**) with the molecular formula C_8_H_7_N, 4-hydroxybenzaldehyde [26] (**2**) with C_7_H_6_O_2_, 1H-indole-3-carboxaldehyde [27,28] (**3**) with C_9_H_7_NO, and (3S,8aS)-3-[(4-hydroxyphenyl)methyl]-2,3,6,7,8,8a-hexahydropyrrolo[1,2-a]pyrazine-1,4-dione (Cyclo (-Pro-Tyr)) [29] (**4**) with C_14_H_16_N_2_O_3_. Compounds **1**–**4** are reported for first time from this strain. NMR spectra are available in Supplementary Material.

### 2.3. In Vitro Acetylcholinesterase, Alphaglucosidase and Xanthine Oxidase Inhibition Assay

The results obtained from the inhibitory activity of AChE, AG and XO corresponding to the extract of *V. neocaledonicus* and the isolated compounds indole; 4-hydroxybenzaldehyde, and 1H-indole-3-carboxaldehyde at different doses are shown in Table 1 and Table 2.

Based on Table 2, the extract of *V. neocaledonicus* showed 98.34% and 69.67% inhibition of AChE statistically significant at the two highest doses 50 and 25 µg/mL, respectively. For the other two enzymes evaluated (AG and XO), percentages of inhibition were also presented in all the doses used although they were not statistically significant. For the isolated compounds, both indole and 4-hydroxybenzaldehyde, showed moderate percentages of inhibition compared with the extract, which suggests that the high inhibitory activity of the extract may be due to a synergy between the components, although the percentages of inhibition of the compounds separately were not significant. In addition, according to the data listed in Table 2, indole showed a strong dose-dependent inhibitory activity against AG enzyme, with 94.4, 85.49, 81.49 and 51.43% inhibition at the doses 25, 12.5, 6.25, and 3.125 µg/mL, respectively. While 4-hydroxybenzaldehyde and 1H-indole-3-carboxaldehyde showed no inhibitory activity for the enzyme AG at any of the tested doses.

In the XO inhibition bioassay, no activity was observed for indole and 4-hydroxybenzaldehyde. However, the activity of 1H-indole-3-carboxaldehyde in all the doses analyzed was very high and significant (100%, 86.49%, 71.29%, 67.69% and 61.14%) (Table 2).

### 2.4. Antiophidic Activities

#### 2.4.1. Inhibition of Proteolytic Activity by Indole

**Figure 3:**
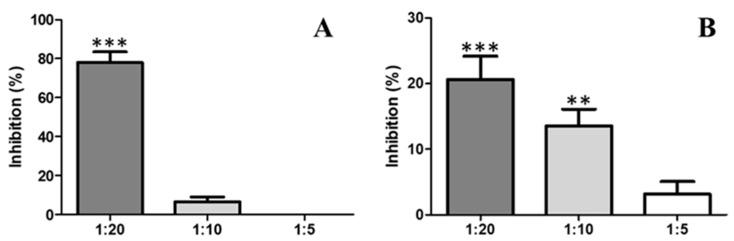
Inhibition of the proteolytic activity of *B. asper* venom (**A**) and a metalloproteinase (**B**) by indole. A dose of 3 µg of venom or toxin per well was used. *** = *p* value < 0.001, ** = *p* value < 0.01 with respect to positive control.

When *B. asper* venom was pre-incubated with indole at varying ratios, inhibition of the proteolytic activity of *B. asper* venom was observed (Figure 3A). The venom:compound ratio of 1:20 showed proteolytic activity inhibition of 77.9% ± 5.5. These differences were statistically significant with respect to the positive control. The venom:compound ratio 1:10 showed weak inhibition without significance.

Likewise, the inhibitory capacity of indole on the proteolytic activity induced by a Metalloprotease isolated de *B. atrox* was evaluated. When the toxin was pre-incubated with indole at varying ratios, a low degree of inhibition of the proteolytic activity was observed (Figure 3B). The inhibition showed dose-dependent behavior, 1:20 (20.61 ± 3.5); 1:10 (13.54 ± 2.5) and 1:5 (4.31 ± 1.9) (Figure 3B). The inhibition at 1:5 was not significant compared to the control.

#### 2.4.2. Inhibition of the Activity Coagulant by Indole

**Figure 4:**
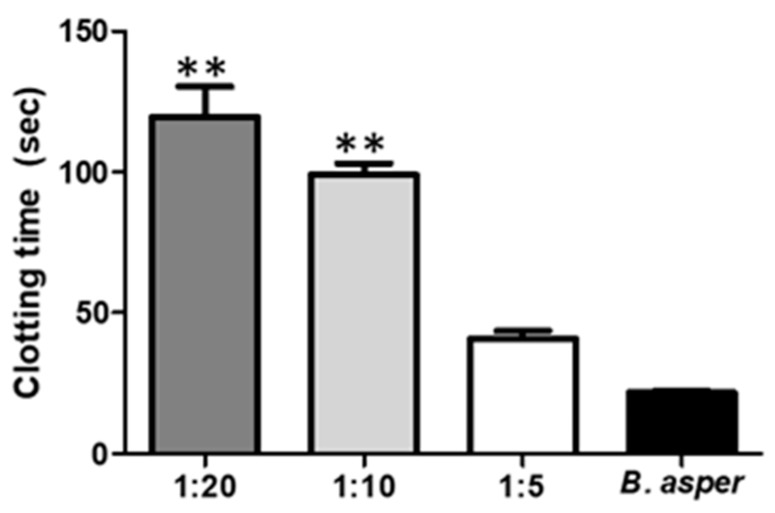
Inhibition of the complete venom coagulant activity of *B. asper* by indole. A dose of 1µg of venom per tube was used. ** = *p* value < 0.01 with respect to positive control.

The compound indole, showed significant inhibition of the coagulating effect in vitro induced by 1.0 µg of venom, corresponding to a clotting time of 21.6 ± 0.8 s. Initially, the coagulant activity was changed at low ratios of 1:5 (venom:compound w/w). However, the extension of the coagulation time became statistically significant when the venom was used at a ratio of 1:10 and 1:20 with indole, with the maximum delay times for clotting on set found to be 119.7 ± 10.7 s and 99 ± 4.0 s, respectively. From this relationship, the inhibitory effect was shown to depend on the compound concentration. Likewise, after half an hour of incubation, it was possible to validate that the compound relations used for this trial did not generate coagulant activity by themselves (Figure 4).

#### 2.4.3. Inhibition of PLA_2_ Activity

**Figure 5:**
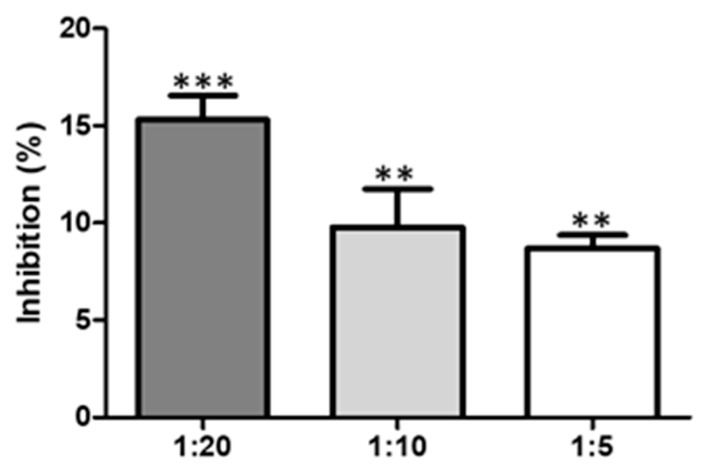
Inhibition of PLA_2_ activity by indole. A dose of 7 µg of pure toxin. There is no significant inhibition against PLA_2_ activity. *** *= p* value *<* 0.001, ** = *p* value < 0.01 with respect to positive control.

To evaluate the activity of PLA_2_, two different techniques were applied: Indirect hemolytic activity and the use of 4-nitro-3-octanoyloxybenzoic acid (4N3OBA) as monodispersed substrate. A low degree of inhibition against PLA_2_ activity by indole was observed (Figure 5). The toxin:compound ratio of 1:20 showed proteolytic activity inhibition of 15.20%; while a ratio of 1:10 showed proteolytic activity inhibition of 10.0%. These differences were not statistically significant between the evaluated ratios, but they were significant with respect to the positive control (Figure 5). Indole did not show inhibition of the indirect hemolytic activity of the complete venom of *B. asper* at tested doses.

### 2.5. Molecular Docking Studies

**Figure 6:**
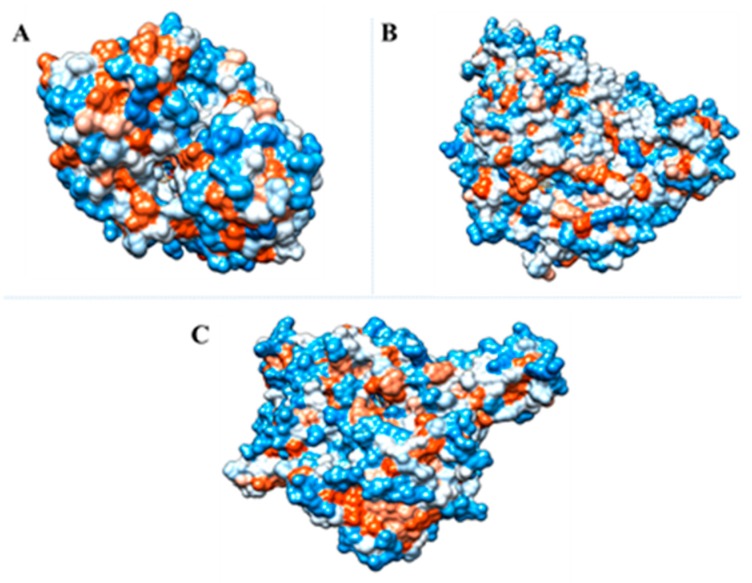
Molecular docking. (**A**) Indole with active site residues of AChE (**B**) Indole with active site residues of AChE (**C**) 1H-indole-3-carboxaldehyde with active site residues of XO.

**Figure 7:**
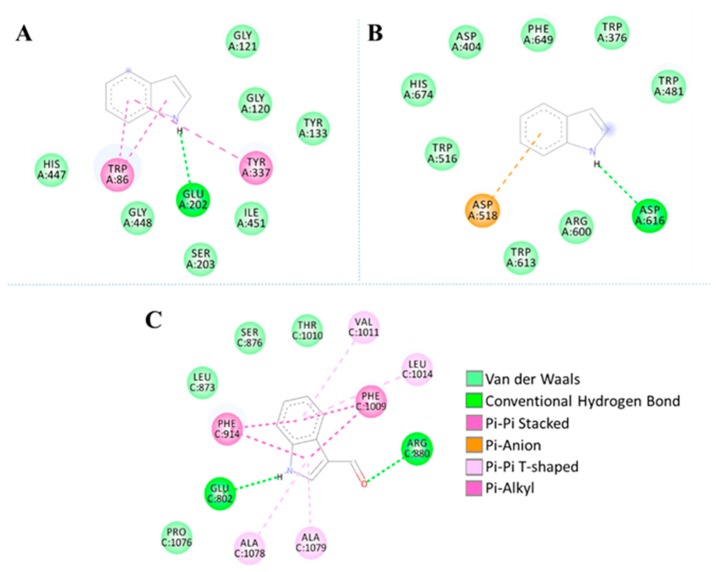
Binding of ligands to active site of studied enzymes. (**A**) Indole + AChE (**B**) Indole + AG (**C**) 1H-indole-3-carboxaldehyde + XO. The red areas of the surface represent the acid regions; the white areas represent the neutral and the blue areas the basic regions.

In order to get the possible mode of inhibition of AChE and AG by indole, and XO by 1H-indole-3-carboxaldehyde, molecular docking simulations were performed. Docked solutions with the lowest binding energies were selected and the results obtained are summarized in Table 3. These results suggest that indole and 1H-indole-3-carboxaldehyde may occupy the AChE, AG and XO active site (Table 3 and Figure 6 and Figure 7).

## 3. Discussion

The chemical study of *V. neocaledonicus* allowed the identification of indole (**1**) as one of the major compounds responsible of the inhibitory activity of AChE and AG. In addition, other three compounds were isolated and identified as 4-hydroxybenzaldehyde (**2**), 1H-indole-3-carboxaldehyde (**3**) and Cyclo (l-Pro-l-Tyr) (**4**), which may contribute to an increase in knowledge of the chemistry on *V. neocaledonicus*.

Despite the narrow structural margin reported for vibrios, compared to metabolites produced by other marine bacteria, vibrios produce compounds with a wide range of interesting biological activities including antibacterial, anticancer and antiviral activities [6]; a clear example being the *V. neocaledonicus* species of the present study. The production of compounds from microorganisms is regulated by many factors, such as the sources of carbon, nitrogen, phosphorus and oxygen in the medium as well as the temperature, pH, and agitation. Such cultivation conditions are a key element for the discovery and production of secondary metabolites as they can activate or deactivate different biosynthetic routes [30]. From this fact, it is of paramount importance to know the ecological functions of these metabolites, as well as the specific conditions in which their biosynthesis is given, in order to understand and to manage the production of those compounds in in vitro conditions [31,32]. There are some examples where the concentration of phosphates and availability of nitrogen sources can affect the production of compounds [33,34]. These qualitative differences among extracts of the same strain can be explained by the variances between the composition of the medium in relation to the content of nitrogen and phosphorus, which then affected the production of metabolites in *V. neocaledonicus* (Figure 1). We found that *V. neocaledonicus* produced indole with a fairly high yield (9.20%) in the culture made with liquid medium 2216E. There are reports that many species of bacteria can produce indole [35], however, despite the high energy cost that this represents for bacteria, it is still not clear why they produce large quantities of this compound.

According to Lee and Lee [35], indole is an intercellular signal chemical in gram positive (+) and gram negative (-) bacteria and has been shown to control a number of bacterial processes such as spore formation, plasmid stability, drug resistance, biofilm formation, and virulence. Moreover, it has been reported that indole can form dimers through carbon-carbon, carbon-nitrogen, or nitrogen-nitrogen linkage to form a small class of biindol marine alkaloids with cytotoxicity against cancer cell lines [36]. Likewise, Atanasova [37], reported the design and prediction of new derivatives of galantamine with the indole molecule in the lateral chain to inhibit the activity of the enzyme AChE. The inhibitory activity AG of indole is comparable with the activity of Acarbose (Table 1 and Table 2) which is one of the drugs used for diabetes. The above indicates the great potential of this compound for the treatment of diabetes. On the other hand, taking into account that this species can produce high amounts of indole under certain growing conditions, it is possible to produce indole by cultivating this strain on a large scale, without causing environmental problems, in a sustainable and economic way. Additionally, the data that support the strong activity of indole against enzyme AG relate to its chemical structure. According to Kim [38], there are three types of inhibitors for AG based on its chemical structure: N-substituted heterocyclic poly-hydroxy; Cycle-alkenes poly-Hydroxyl and Oligomers pseudo sugars. Indole is an N-substituted aromatic heterocyclic organic compound. At a pharmacological level, within the natural inhibitors of this enzyme, there are compounds of bacterial origin such as acarbose, miglitol and voglibose (hemi-synthetic) [39], and these are active ingredients in anti-diabetes drugs for type II diabetes [40].

4-hydroxybenzaldehyde (**2**), an analog of p-hydroxybenzyl alcohol, showed a slight inhibitory effect on AChE and XO. Its antiepileptic and anticonvulsant activity has been reported as being even greater than that of valproic acid, a known anticonvulsant [41]. In addition, 4-hydroxybenzaldehyde derivatives have been synthesized and investigated for their inhibitory effects on fungal tyrosinase and Gamma Amino-Butyric Acid GABA-derived enzymes [42]. 1H-indole-3-carboxaldehyde (**3**) exhibited a very strong inhibitory activity to XO (IC_50_ 13.36 ± 0.39 µM). The results obtained from this study are comparable with other known XO inhibitors such as Allopurinol, an analogue of hypoxanthine for the treatment of gout, and other compounds with the same activity reported such as Febuxostat. In spite of the fact that the mechanism of action of indole-3-carboxaldehyde is not well understood, it is interesting to analyze the mechanism of action of allopurinol (our positive control) to try to give an application to the action of this compound (**3**). First, Allopurinol acts as a substrate and subsequently as an inhibitor of XO. The oxidase hydroxylates allopurinol to form alloxanthin, which is then tightly bound to the active center. The binding of alloxanthin keeps the molybdenum atom of the enzyme in a +4 oxidation state, preventing it from returning to the +6 oxidation state, just as it would in a normal catalytic cycle. This type of inhibition is known as "suicidal inhibition" and could be a hint to elucidate the mechanism of action of indole-3-carboxaldehyde (**3**). Interestingly, some indole-3-carboxaldehyde derivatives have been reported in terms of synthesis and antimicrobial activity (antibacterial, antifungal and antiamebian) [43,44].

The compound Cyclo (L-Pro-L-Tyr) (**4**) was not evaluated for bioactivities due to the low quantity obtained; however, this compound is reported for the first time for the strain. In addition, small molecules isolated from the species of genus Vibrio that induce gram-negative quorum sensing systems (QS) detection have been reported, and some of the molecules possess a group of diketopiperazines (DKP), for example, cycle (L-Pro, L-Leu), cycle (L-Pro, L-Val) and cycle (L-Pro, L-Tyr) [45]. On the one hand, it is likely that these dipeptides represent a new class of naturally occurring QS signals that can participate in interspecific signaling, since DKPs are found in most culturable marine bacteria [46]; however, it is also possible that some DKPs are generated from media components during processing procedures [47].

Snake venom is designed to immobilize, kill and help digest prey. It is made up of different toxins that can be grouped into a small number of major protein families, including phospholipase A_2_ (PLA_2_), zinc-dependent metalloproteinases, serineproteases, C-type lectin proteins, disintegrins, cysteine rich proteins (CRISPs), bradykinin potentiating peptides and l-aminoacidoxidase, and others [48]. Consequently, viper snakebites characteristically cause local and systemic pathological changes, such as coagulation disorders, systemic hemorrhage, necrosis, thrombocytopenia, pain and edema, which may vary in intensity depending on the species, age and size of snake [48,49]. The neutralization of all these effects by anti-venoms is a difficult task. However, the microorganisms represent potential sources of inhibitors and could be an alternative for tackling this problem. In this study, indole isolated from *V. neocaledonicus*, showed an inhibitory capacity to the toxic activities of *B. asper* venom in an in vitro model. *B. asper* venom affects blood coagulation by allowing the formation of fibrin from fibrinogen due to the presence of toxins that activate platelets and factor XII, whereas the molecular factors V and VI found in the venom directly activate factor X. As fibrinogen is converted to fibrin, it becomes more unstable and susceptible to lysis by natural fibrinolytic systems [20]. As shown in Figure 4, indole showed a greater extension of the clotting time, the delay in coagulation was effective in neutralizing the coagulating effect when administered in a ratio of 1:10 and 1:20 (119.7 ± 10.7 s and 99 ± 4.0 s respectively). So, indole has the potential to interact with serine proteinases; these enzymes are present in the venom and are responsible for its plasma coagulation effect. Certainly, indole also showed some degree of inhibition of other activities of the venom such as proteolytic activity (Figure 3A,B) and hemolytic activity (Figure 5). Although the results were not statistically significant, it is important to mention that the compound has the ability to interact with the toxins responsible for these effects (Metalloprotease and PLA_2_).

The experimental results in the in vitro model correlate well with the molecular coupling data results (Figure 6 and Figure 7). Figure 6A showed that hydrogen bond and π − π stacked interactions between indole and AChE could block binding of the quaternary trimethylammonium tail group of ACh, given that the “anionic” subsite of the enzyme is formed by side chains of Glu202, Trp86 and Tyr337. Similarly, π − π stacked interactions with Gly121 could block the oxyanion hole [50]. The active site of all xanthine-utilizing enzymes has a conserved arginine residue (Arg880 in the bovine enzyme), and it has been proposed that this residue contributes to transition state stabilization. On the other hand, Glu802 facilitates xantine tautomerization and thus contributes to rate acceleration [51]. Thus, hydrogen bonds between Arg880, Glu802 and 1H-indole-3-carboxaldehyde could negatively affect enzymatic catalysis (Figure 6C, Table 2).

It has been reported that AG uses a double-displacement reaction mechanism in which aminoacids Asp518 and Asp616 act as catalytic nucleophile and acid/base [52]. Thus, according to Figure 6B, hydrogen bonds between indole with Asp616, and π -anion interactions between indole with Asp518, could inhibit the catalytic cycle of AG. Otherwise, Van der Waals interactions involving Indole and the amino acids that belonged to the hydrophobic pocket, such as, Trp376, Trp516, and Trp613 [53] could enhance the inhibitory effect (Figure 6B, Table 2).

These results suggested the potential feasibility of *V. neocaledonicus*. It is important to focus efforts on the search for AChE, XO and AG inhibitors from natural sources; perhaps several potential inhibitors in marine bacteria are waiting to be discovered to provide easily manipulated natural sources for the mass production of these therapeutic compounds. This fact indicates that the marine organisms have a high potential for the discovery of new and valuable compounds with diverse grade of pharmaceutical applications.

## 4. Materials and Methods

### 4.1. Sample and Reagents

Acetylcholinesterase from *Electrophorus electricus*, alphaglucosidase from *Saccharomyces cerevisiae*, and xanthine oxidase from bovine milk were obtained from Sigma Aldrich (Shanghai, China). The other reagents used in the bioassays were purchased either from Solarbio or Sigma Aldrich. Analytical reagent grade organic solvents for extraction were purchased from XL Xilong Scientific Chemical. Silica gel plates (Silica gel 60 F_254_ 0.2 mm layer thickness) were purchased from Merck (KGaA, Darmstadt, Germany). A spectrometer (Thermo Scientific Multiskan GO, Vantaa, Finland) was used for all measurements of AChE, AG and XO inhibitory activities. The venom was extracted and collected manually from the adult specimens of *B. asper* snakes from Colombia (South America) that were held captive in the Serpentarium at University of Antioquia. The venom was centrifuged, and the supernatant was lyophilized and frozen at −70 °C until its use.

Column chromatography was carried out on silica gel (50–80 Size A° Qingdao Bangkai Hi-tech Materials, Shandong, China) and TLC (TLC Silica gel 60 F_254_ aluminum sheets) was used to monitor the fractions from the column chromatography. Preparative TLC was carried out on glass sheets (Silica gel GF_254_ 20 × 20 cm, 0.9–1.0 mm Qingdao Bangkai Hi-tech Materials). Visualization of the TLC plates was achieved by using a UV lamp (λ¼254 and 365 nm) or/and spraying p-Anisaldehyde (Aladdin Industrial Corporation, Hangzhou, China) reagent then heating at 105 °C for 1 min. ^1^H and ^13^C NMR spectra were measured on a Bruker AMX 500 MHz NMR spectrometer with standard pulse sequences, operating at 500 MHz for ^1^H and 125 MHz for ^13^C.

### 4.2. Marine Bacterial Strain

*V. neocaledonicus* was obtained using the traditional methods of farming. The strain was isolated from coral reef in South China Sea, and characterized and identified using both conventional molecular methods (16S rRNA gene sequencing) based on its morphological characteristics. The voucher number for the organism is CCTCC M2017802. The isolated strain was preserved in a marine medium Difco 2216 semi-solid at 4 °C until it was used for cultivation.

In this study, two types of cultures using the same strain (*V. neocaledonicus*) were made with two different media. The first crop was made with *V. neocaledonicus* cultivated (8.0 L) in 2216E Liquid Medium (Qingdao Hope Bio-Tecnhology Co, Shandong, China) for 10 days at 30 °C with stirring at 160 rpm. The 2216E culture medium (37.4 g/1000 mL of distillated water) was subjected to autoclaving. For the second culture, *V. neocaledonicus* was cultivated (16 L) with LB medium, in which 10.0 g of Tryptone (LP0042 – OXOID), 5.0 g of Yeast extract (LP0021 – OXOID) and 20.8 g of NaCl, (A.R. Guangzhou chemical reagent factory, Guangzhou, China) were used. The bacteria were left to grow for 10 days at 30 °C with stirring at 160 rpm. The water used was distilled through equipment AXL Water. All means and instruments used in microbiology were sterilized with steam in an autoclave (Zealway autoclave-GI54DW, 32 L) under 20 psi and at 121 °C for 20 min. The innocuous ones were prepared in cabin flow laminar ZJ (SW-CJ-1D). Incubation of agar plates was conducted in a ZHWY-2112B Incubator shaker at 30 °C.

### 4.3. Extraction Process

For each of the two crops of *V. neocaledonicus* in two different media, the following procedure was carried out for extraction: biomass was separated by centrifugation at 8000 rpm for 10 min at 29 °C on a centrifuge (Thermo Scientific Heraeus Multifuge X3R, Osterode am Harz, Germany). Subsequently, the supernatant was filtered out and the strain was subjected to maceration with ethyl acetate (1 L × 3 times), then concentrated by using an IKA-HB10 rotary-evaporator under reduced pressure in with a compact vacuum pump MVP 10. The extracts were evaluated using the bioassays for AChEI, AGI and XOI activity.

### 4.4. Isolation of Secondary Metabolites from Vibrio neocaledonicus

For the first culture, ethyl acetate extract (1.2 g) was fractionated by column chromatography (CC) (3 × 50 cm) on silica gel, eluting sequentially with the following solvents mixtures: hexane, hexane-ethyl acetate (7:3), hexane-ethyl acetate (5:5), ethyl acetate, ethyl acetate-methanol (7:3), ethyl acetate-methanol (1:1), methanol. The following spray reagents were used in order to develop the spots: anisaldehyde-sulphuric acid (sterols, phenolic compounds, terpenes), also the plates were revealed with reagent of Draggerdorf to look for presence of alkaloids. According to the behavior on the TLC, the elutes were grouped into eight fractions (F1 to F8): F1 (0.008 g), F2 (0.002 g), F3 (0.198 g), F4 (0.017 g), F5 (0.019 g), F6 (0.027 g), F7 (0.198 g), and F8 (0.531 g). Fraction F3 was separated by CC on silica gel eluting with hexane-acetone (8:2). Subfraction F3.2 (128.8 mg) was further separated by CC eluting with hexane-acetone from 9:1 to 1:1 to offer compound 1 (110.0 mg). Fraction F1 (0.008 g) no color, not active resulted in AChI and AgI bioassay. Fraction F2 (0.002 g) no color, not active resulted in AChI and AgI bioassay. Fraction F3 (0.198 g) brown color, showed activity in AChI and AgI bioassay, was fractionated. We obtained seven sub-fractions (F3.1-F3.7), being the fraction F3.2 (128.8 mg) with strong activity AChI and was fractionated by CC. We obtained eight sub fractions (3.2.1-3.2.8) being the fraction 3.2.2 (57.4 mg) and 3.2.3 (52.7 mg) active in AChI and AgI bioassay. A part of fraction 3.2.2 (3.2 mg) was sent to analyze by NMR. Fraction F4 (0.017 g) in a brown color showed no activity in AChI but strong activity in the AgI bioassay. A CPP was made and three subfractions were obtained. Subfraction 4.1 and 4.2 were send for NMR analysis. Fraction F5 (0.019 g) presented no activity in AChI but was active in AgI bioassay. Then a CPP was made. We obtained five fractions (F5.1–F5.5). Subfraction 5.1, 5.2 and 5.3 were sent for NMR analysis. Fraction F6 (0.027 g) in a dark brown color showed low activity in AChI but strong activity in the AgI bioassay. It was fractionated. We obtained four fractions (F6.1–F6.4), subfraction F6.2 (15 mg) was fractionated and we obtained three fractions F6.2.1–F6.2.3. The subfraction 6.2.3 was fractionated by C.C with hexane-acetone. Fraction F7 (0.198 g) in a dark color presented good activity in AChI and low activity in AgI bioassay. Was fractionated by C.C. We obtained three fractions (F7.1-F7.3); making the fraction 7.3 (61.3 mg) with good activity AChI. Fraction 7.3 was fractionated and five sub fractions were obtained (7.3.1–7.3.5) making the fraction 7.3.2 (1.5 mg), which was active in the AChI bioassay and was sent to be analyzed by NMR. Fraction 7.3.4 (30 mg) was active in the AChI bioassay. Fraction F8 (0.531 g) in a dark color, presented good activity in AChI and low activity in the AgI bioassay. Fraction F8.4 (100.3 mg) was active in AChI and was fractionated. We obtained three sub fractions (8.4.1–8.4.3) being the fraction 8.4.3 (28.7 mg) active in AChI and was purified by CCP. Subfractions 8.4.3.1A, 8.4.3.1B and 8.4.3.1C were active in AChI bioassay and they were sent for NMR analysis.

For the second culture, ethyl acetate extract (3.6 g) was fractionated by CC on silica gel eluting sequentially with hexane, hexane-ethyl acetate from 9:1 to 1:1, ethyl acetate, ethyl acetate-methanol (9:1), ethyl acetate-methanol (7:3), ethyl acetate-methanol (1:1) and methanol. According to the behavior on the TLC, the elutes were grouped into seven fractions (F1 to F7): F1 (0.0202 g), F2 (0.0047 g), F3 (0.180 g), F4 (0.030 g), F5 (0.025 g), F6 (0.091 g) and F7 (1.7 g). Fraction F5 was purified by using a preparative TLC plate with hexane-acetone (7:3) as mobile phase to give compound 2 (6.5 mg). Fraction F6 (91.0 mg) was separated by CC on silica gel eluting with a mixture of hexane-acetone from 7:3 to 1:1, then acetone and methanol. Subfraction F6.2 (53.0 mg) was fractionated by CC eluting with hexane:acetone from 7:3 to 1:1 to give subfraction 6.2.1 (35.2 mg), which was subjected to being purified on a preparative TLC plate with hexane-acetone 6:4 as mobile phase to afford compound 3 (10.0 mg) of. Fraction F7 (1700 mg) was separated by CC on silica gel eluting with dichloromethane-methanol from 9:1 to 1:1 to give the subfraction 7.5.1 (12.2 mg), which was further purified on a preparative TLC plate with dichloromethane-methanol 9:1 to provide compound 4 (4.3 mg).

^1^H and ^13^C NMR spectra were obtained on a Bruker model AMX 500 MHz NMR spectrometer with standard pulse sequences. The chemical shift values were reported in parts per million units (ppm) from tetramethyl silane as standard reference. Coupling constants were calculated in hertz (Hz). The 2D NMR spectra including COSY, HMQC and HMBC were measured using the standard pulse sequences.

### 4.5. Acetylcholinesterase Inhibition Assay

The extracts and compounds 1–3 isolated from *V. neocaledonicus* were submitted for bioassay. AChE inhibition was determined according to the method of Ellman [54] with ATCI as substrate. In short, to a 96-well microplate, 20 μL of 0.30 mg/mL ATCI, 10 μL of different concentrations (ranging from 3.125–50 μg/mL) of sample solution (extract or compound dissolved in 2% of DMSO and PBS), 10 μL of 1.0 μg/mL AChE, 40 μL of 0.02 mol/LPBS (pH 7.4), after incubation at 37 °C for 30 m, 20 μL 4% SDS was added to stop the reaction. Then, 100 μL of 0.59 mg/mL DTNB was added to produce the color, the OD was measured at 405 nm in spectrometer. All determinations were performed in triplicate with Huperzine as positive control. The control contained all components except the tested extract. The inhibitory rate was calculated as the formula: %I = [1 − (ODs−OD sample sb)/ODn-ODnb] × 100%; where: ODn is the absorbance value of the sample system in which sample is substituted by PBS, ODnb is the absorbance value of ODn in which no AChE was added, ODsb is the absorbance of ODs in which no AchE was added.

### 4.6. Alphaglucosidase Inhibition Assay

Extracts and compounds 1–3 isolated, from *V. neocaledonicus*, were submitted for bioassay. The AG inhibition activity was evaluated by measuring the release of p-nitrophenol from p nitrophenyl-α-d-glucopyranoside (pNPG) following the protocol proposed by Pistia-Brueggemann [55], with modifications. The assay contained 80 μL of phosphate buffer (PBS 0.5 M, pH 6.8); 10 μL of l-Glutathione (reduced-Solarbio Life Sciences) solution to protect the enzyme, 20 μL of enzyme, 20 μL of α-glucosidase (1 UI/mL, derived from *S. cerevisiae*); 20 μL of different concentrations (ranging from 3.125–50 μg/mL) of sample solution (extract or compound dissolved in 2% of DMSO and PBS); 40 μL of pNPG (11.3 mM). The control (C-) contained all the reagents but the sample was substituted by PBS. All determinations were carried out in triplicate with Acarbose as positive control. The absorbance was read after incubation for 30 min at 37 °C in a spectrometer Thermo Scientific Multiskan GO reader at 405 nm. The inhibitory rate was calculated as the formula: %I = [1 − (ODs − OD sample sb)/ODn-ODnb] × 100%; where: ODn is the absorbance value of the sample system in which sample is substituted by PBS, ODnb is the absorbance value of ODn in which no AG was added, ODsb is the absorbance of ODs in which no AG was added.

### 4.7. Xanthine Oxidase Inhibition Assay

Extract and compounds 1–3 isolated, from *V. neocaledonicus*, were submitted for bioassay. The assay was based on the developed method of Valentao [56] and modified for application in microplates by Lopez-Cruz [57]. All test samples were dissolved in a 50 mM phosphate buffer to simulate the environment in which the reaction occurs in the body. Different concentrations (ranging from 3.125–50 μg/mL) of sample solution (extract or compound dissolved in 2% of DMSO and PBS). Finally, 0.5 U/mL of enzyme XO solution was added and the absorbance was recorded at 295 nm. Allopurinol 1.2 mmol /L was used as a positive control. The control contained all components except the sample. The inhibitory rate was calculated as the formula: %I = [1 − (ODs − OD sample sb)/ODn-ODnb] × 100%; where: ODn is the absorbance value of the sample system in which sample is substituted by PBS, ODnb is the absorbance value of ODn in which no XO was added, ODsb is the absorbance of ODs in which no XO was added.

### 4.8. Antiophidic Activity

#### 4.8.1. Purification of the Metalloproteinase (Batx-I) and Phospholipase A_2_ (Cdcum6)

Batx-I was isolated from *Bothrops atrox* according to the protocol of Patiño [58]. Purification was done by ion-exchange chromatography (CM Sephadex C25) and the toxin purity was tested by RP-HPLC and SDS PAGE. PLA_2_ was isolated from *Crotalus durissus cumanensis* according to the protocol of Pereañez [59]. PLA_2_ was purified by RP-HPLC on C-18 column eluted at 1.0 mL/min with a gradient from 0% to 100% acetonitrile in 0.1% trifluoroacetic acid (v/v). Absorbance was monitored at 215 nm. Batx-I and Cdcum6 were lyophilized and stored at −20 °C until their use.

#### 4.8.2. Inhibition of PLA_2_ Activity

The inhibitory effect of indole on the PLA_2_ of snake venom was assessed by two methods; the first of them, using monodispersed substrate 4N3OBA (manufactured by BIOMOL, Hamburg Germany) according to the method described by Holzer and Mackessy [60] and adapted for a 96-well ELISA plate. The standard assay contained 200 µL of buffer (10 mM Tris–HCl, 10 mM CaCl_2_, 100 mM NaCl, pH 8.0), 20 µL of 10 mM of substrate (4NO3BA), 20 µL of sample (20 µg PLA_2_ or 20 µg PLA_2_ + several concentrations of indole) and 20 µL of water. The negative control was only buffer. The inhibitory effect of indole on PLA_2_ activity was determined through pre-incubation of the enzyme with each concentration of the compound for 30 min at 37 °C. After the incubation period, the sample was added to the assay and the reaction was monitored at 425 nm for 40 min at 37 °C. The quantity of chromophore released (4-nitro-3-hydroxy benzoic acid) was proportional to the enzymatic activity. The results are indicated as inhibition percentages.

The second method to evaluate the inhibitory effect of indole on PLA_2_, was determining the inhibition of the indirect hemolytic activity. This was evaluated in agarose-erythrocyte-egg yolk gels according to Gutierrez [61]. It used a minimum indirect hemolytic dose (MIHD) of *B. asper* venom of 2.2 μg, defined as the amount of venom that induced a 20 mm diameter hemolytic halo. Venom and different doses of indole were preincubated for 30 min at 37 °C and the inhibitory potential was measured after 20 h of plate incubation at 37 °C. This experiment was done in triplicate.

#### 4.8.3. Inhibition of Coagulant Activity

The method described by Theakston and Reid [62] was followed, with small modifications. Briefly, 1.0 µg of *B. asper* dissolved in 25 µL PBS were mixed with 75 µL of different concentrations of indole, and pre-incubated for 30 min at 37 °C. After, 100 µL of mixtures were added to 0.2 mL of plasma, and the time required for plasma coagulation was determined.

#### 4.8.4. Inhibition of Proteolytic Activity

The proteolytic activity was measured on azocasein (Sigma Aldrich, St. Lous, MO, USA) according to Wang [63] with some modifications. Briefly, 3 µg of *B. asper* venom or Batx-I were dissolved in 5 μL of 25 mM Tris (0.15 M NaCl, 5 mM CaCl_2_), pH 7.4, and were mixed with different doses of indole in order to obtain 1:20, 1:10 and 1:5 w/w ratios, respectively. Mixes were pre-incubated for 30 min at 37 °C. Later, these solutions were incubated with 10 mg/mL of azocasein diluted in the same buffer. After an incubation of 90 min at 37 °C, the reaction was stopped by the addition of 200 μL of trichloroacetic acid. The samples were then centrifuged at 3000 rpm for 5 min. The supernatant (100 μL) was mixed with an equal volume of 0.5 M NaOH, and the absorbance was measured at 450 nm. Results are shown as percentage of activity (absorbance at 450 nm). The assay was carried out in triplicate.

### 4.9. Molecular Docking Studies

The software Avogadro 1.2 [64] was used to build the indole and 1H-indole-3-carboxaldehyde and to improve its overall structure by an energy minimization process based on the MMF94 force field by means of a steepest-descent algorithm in 500 steps. Molecular docking was carried out on a personal computer using AutodockVina [65]. The protein pdb codes were 4PQE, 5NN8 and 3NVY for AChE, AG and XO, respectively. The structure was refined on the WHAT IF package [66] to correct errors from the experimental output such as missing atoms and undesirable location of side chains. Proteins were used without water molecules. The structures of the proteins were prepared using the Protein Preparation module implemented in the Maestro program. First, hydrogen atoms were automatically added to each protein according to the chemical nature of each amino acid, based on the ionized form expected in physiological condition. This module also controls the atomic charges assignment. Second, each 3D structure of the protein was relaxed through constrained local minimization, using the Optimized Potentials for Liquid Simulations (OPLS) force fields to remove possible structural mismatches due to the automatic procedure employed to add the hydrogen atoms. The grid size for each receptor was 24 Å^3^ and the grid centers were X = 33.6, Y = 19.8, Z = 12.3; X = −28.2, Y = −23.8, Z = 1.4; X = −18.8, Y = −24.5, Z = 99.6 for 3NVY, 4PQE and 5NN8, respectively. The receptor grid was generated by identification of active site residues. Exhaustiveness = 20. Then, the ligand poses with best affinity were chosen, and a visual inspection of the interactions at the active site was performed and recorded. The open functionalities of the Discovery Studio 4.0^®^ package by Accelyrs and UCSF Chimera (www.cgl.ucsf.edu/chimera/) were used to generate docking images.

### 4.10. Statistical Analysis

In order to determine significant differences among the concentrations of extract or compound used in the enzymes inhibition assays, the results were analyzed using one-way ANOVA non parametric Kruskal Wallis with Bonferroni post-test. Significant differences were considered when the p values were at least *p* ≤ 0.001. Results are shown as mean x ± SEM of n indicated in each case.

## 5. Conclusions

In conclusion, we present here a study to identify bioactive compounds isolated from marine bacterium *V. neocaledonicus* and their enzyme inhibitory activities. The compounds isolated from this species, particularly indole, are potential inhibitors of enzymes. The results of the molecular coupling for the isolated main compound (indole) show the interesting inhibitory potential to AChE, AG, XO and myotoxic PLA**_2_**_s_. More tangible evidence of its usefulness is required, such as the design and testing of derivatives, whose results could guide the development of an AChE inhibitor. Our results suggest that indole could interact with the active site and other regions of the AChE and AG enzymes, blocking its catalytic cycle and substrates binding. Additionally, this is the first comprehensive report of a chemical study of *V. neocaledonicus* in China. Future studies should be related to the continuation of the isolation of the secondary metabolites of *V. neocaledonicus*, in addition to the exploration and elucidation of the mechanism of action of the compound or compounds responsible for the evaluated activities. In the future, it might be important to extend the sequencing of the vibrios complete genome and to investigate the prevalence of biosynthetic genes linked to secondary metabolism to contribute, in general, to the knowledge of the ecological roles of these bacteria and the environmental and physiological factors that regulate the production of their secondary metabolites.

## Figures and Tables

**Table 1:** IC_50_ value of acetylcholinesterase (AChE), alpha-glucosidase (AG) and xanthine oxidase (XO) inhibitory activity of *V. neocaledonicus* extract and isolated compounds indole, 4-hydroxybenzaldehyde, and 1H-indole-3-carboxaldehyde. AChEI—acetylcholinesterase inhibitors.

Group	AchE (IC_50_ µM)	AG (IC_50_ µM)	XO (IC_50_ µM)
Huperzine (positive control)	0.445	-	-
Acarbose (positive control)	-	387.21 ± 12.54	-
Allopurinol (positive control)	-	-	7.82 ± 0.12
*V. neocaledonicus* extract	15.27 ± 0.07 µg/mL	53.52 ± 1.4 µg/mL	341 ± 2.53 µg/mL
Indole	334.81 ± 13.12	18.65 ± 1.1	842.58 ± 16.73
4-hydroxybenzaldehyde	6892.25 ± 117.58	-	506.23 ± 16.01
1H-indole-3-carboxaldehyde	-	-	13.36 ± 0.39

**Table 2:** Percentage inhibitory activities, on AChE, AG and XO corresponding to the extract of *V. neocaledonicus* and three isolated compounds at different doses: Indole, 4-hydroxybenzaldehyde and 1H-indole-3-carboxaldehyde.

Group	Dose (µg/mL)	AChEI%	AGI%	XOI%
*V. neocaledonicus* extract	50	98.34 ± 0.52 ***	47.28 ± 0.18	26.11 ± 0.03
	25	69.67 ± 0.6 **	33.33 ± 0.95	25.54 ± 0.13
	12.5	22.34 ± 0.56	31.77 ± 0.45	24.62 ± 0.2
	6.25	15.1 ± 0.11	29.06 ± 0.26	22.49 ± 0.03
	3.125	6.23 ± 0.1	8.24 ± 0.03	7.62 ± 0.17
Indole	50	51.3 ± 1.67 *	100 ***	44.30 ± 1.14
	25	37.54 ± 1.26	94.4 ± 1.01 ***	25.17 ± 0.65
	12.5	36.63 ± 1.04	85.49 ± 0.83 ***	22.07 ± 0.55
	6.25	9.42 ± 0.07	81.49 ± 1.17 ***	17.77 ± 0.52
	3.125	0	51.43 ± 1.06 *	15.01 ± 0.31
4-hydroxybenzaldehyde	50	25.7 ± 0.56	0	50.75 ± 0.50
	25	14.26 ± 0.011	0	31.67 ± 0.52
	12.5	6.55 ± 0.02	0	27.28 ± 0.27
	6.25	4.21 ± 0.01	0	25.27 ± 0.19
	3.125	0	0	17.39 ± 0.49
1H-indole-3-carboxaldehyde	50	0	0	100 ***
	25	0	0	86.49 ± 0.89 ***
	12.5	0	0	71.29 ± 0.99 ***
	6.25	0	0	67.69 ± 0.34 **
	3.125	0	0	61.14 ± 0.14 **

Significant differences were considered when the *** = *p* value < 0.001, ** = *p* value < 0.01, * = *p* value < 0.05 with respect to control.

**Table 3:** Interactions and affinities of indole with active-site residues of the AChE and AG, and 1H-indole-3-carboxaldehydewith active-site residues of the XO.

Enzyme	Ligand	Interactions						Affinity (Kcal/mol)
		Hydrogen bonds	Van der Waals	π − π stacked	π-anion	π − π T-shaped	π-alkyl	
AChE (PDB code 4PQE)	Indole	Glu202	Gly120 Gly121 Tyr133 Ser203 His447 Ile451 Gly448	Trp86 Tyr337	-	-	-	−6.3
AG (PDB code 5NN8)	Indole	Asp616	Trp376 Asp404 Trp481 Trp516 Trp613 Arg600 His647 Phe649	-	Asp518	-	-	−5.5
XO (PDB code 3NVY)	1H-indole-3- carboxaldehyde	Arg880 Glu802	Leu873 Ser876 Thr1010 Pro1076	-	-	Phe914 Phe1009	Val1011 Leu1014 Ala1078 Ala1079	−7.2

## Supplementary Materials

The following resources are available online at https://www.mdpi.com/1660-3397/17/7/401/s1, Figure S1: NMR Indole, Figure S2: NMR 4-hydroxy-benzaldehyde, Figure S3: NMR 1H-indole-3-carboxaldehyde.
