# Supplementary material for: Bioactive Compounds Isolated from Marine Bacterium Vibrio neocaledonicus and Their Enzyme Inhibitory Activities
Source: Mar Drugs. 2019 Jul 8;17(7):401. doi: 10.3390/md17070401 (PMC6669558; doi:10.3390/md17070401)
Supplement: Supplementary file 1 [file marinedrugs-17-00401-s001.pdf]

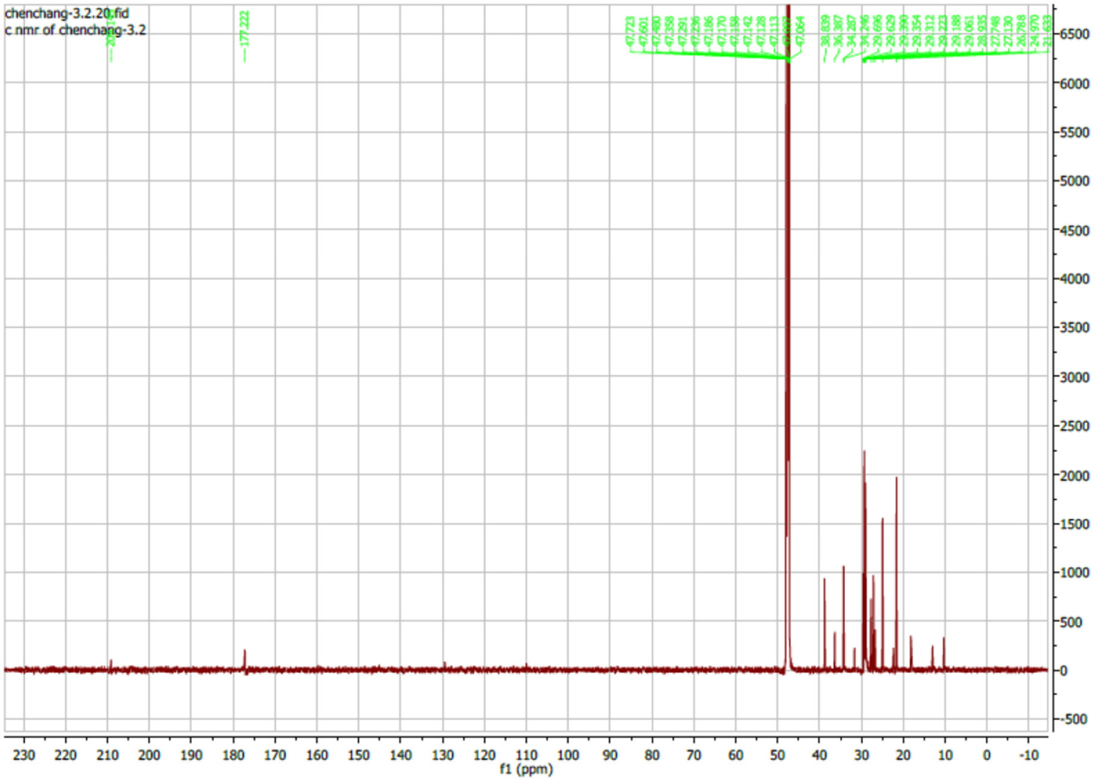
chenchang-3.2.20.fid
c nmr of chenchang-3.2
f1 (ppm)

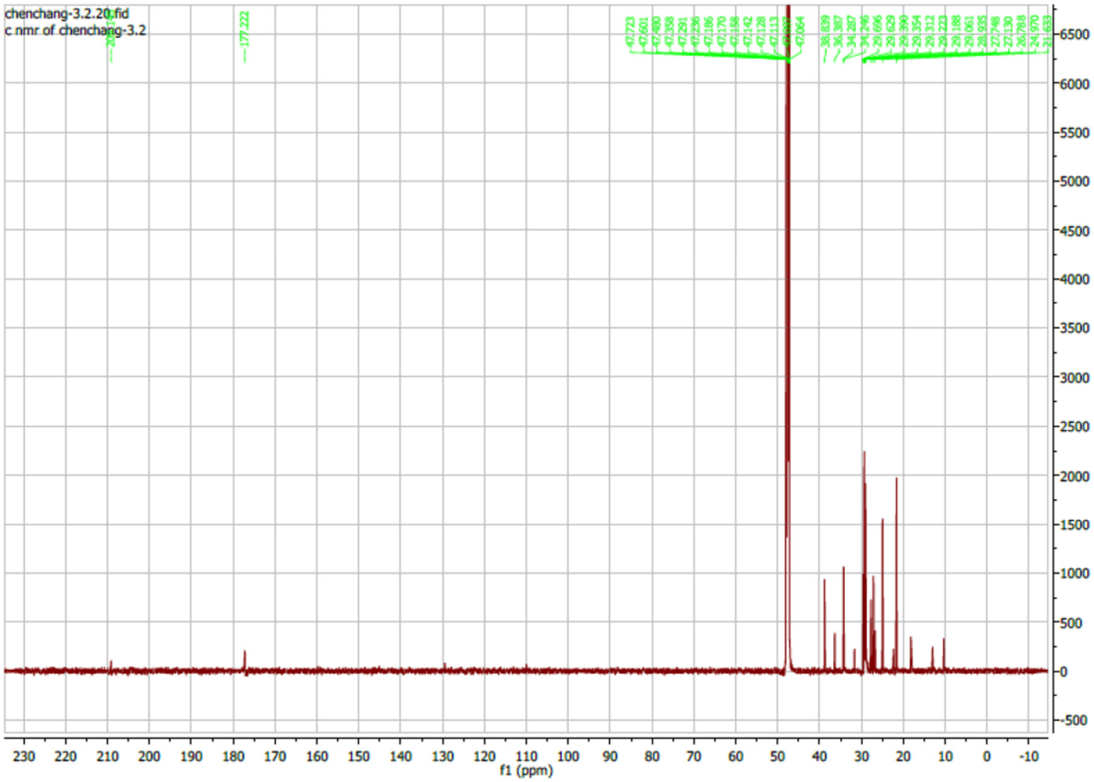
chenchang-3.2.20.fid
c nmr of chenchang-3.2
f1 (ppm)

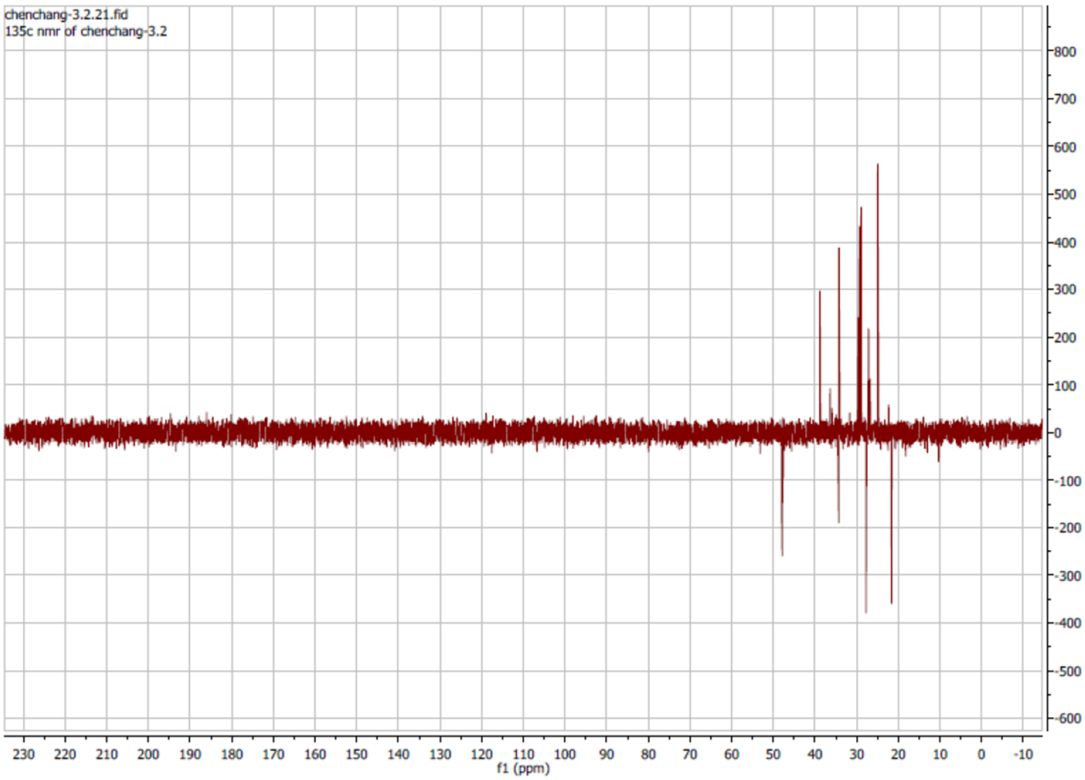

Figure S1. NMR Indole.

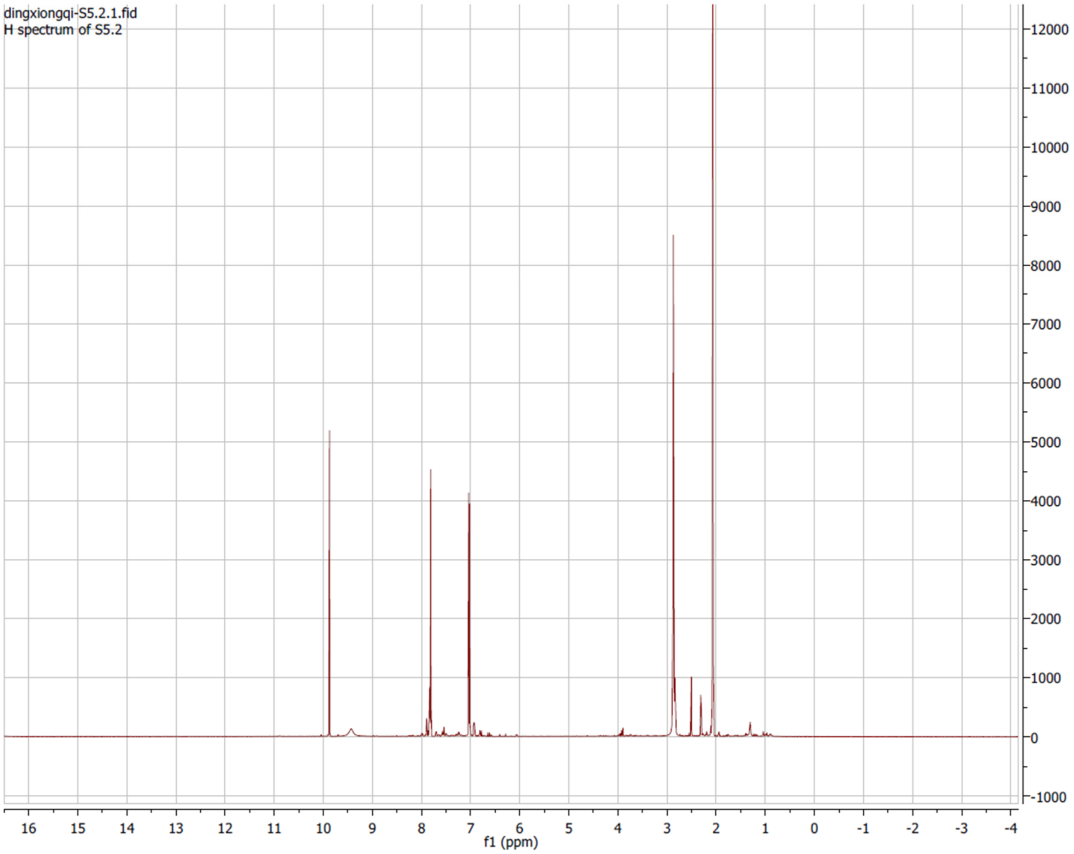

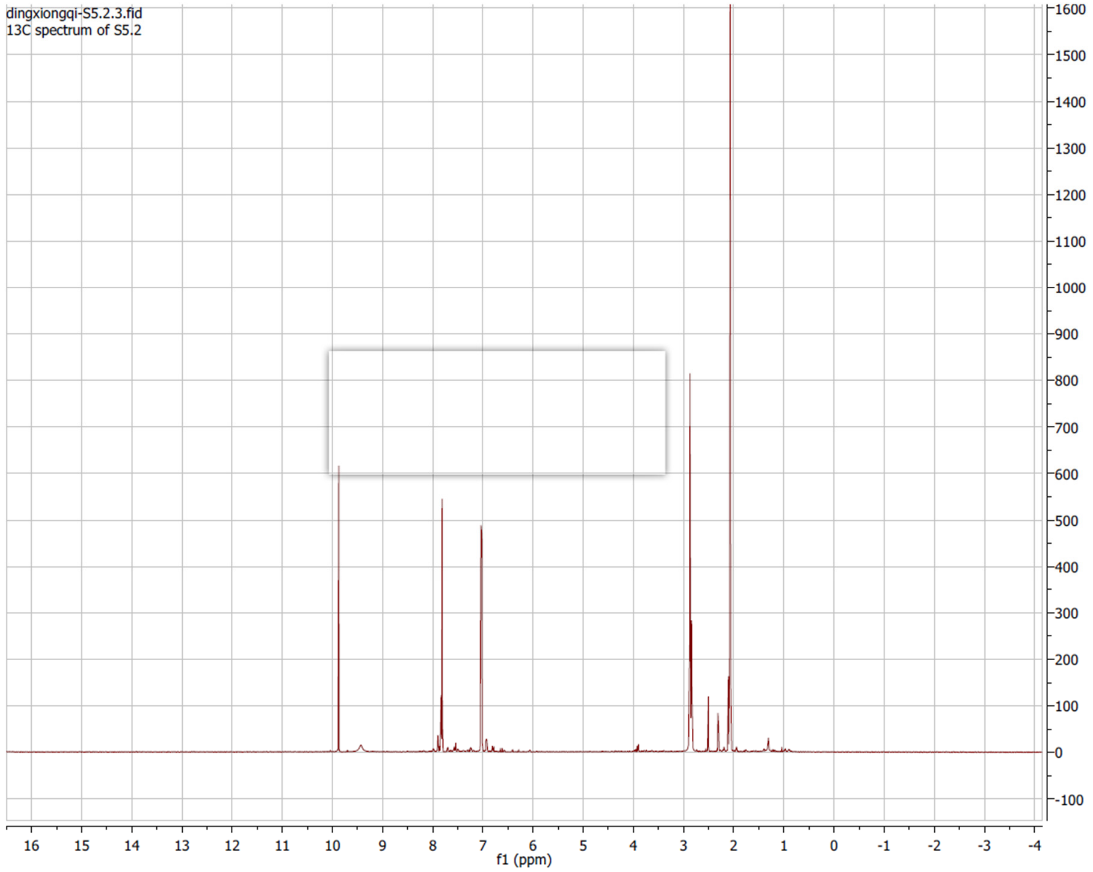
dingxiongqi-S5.2.3.fid
13C spectrum of S5.2
f1 (ppm)

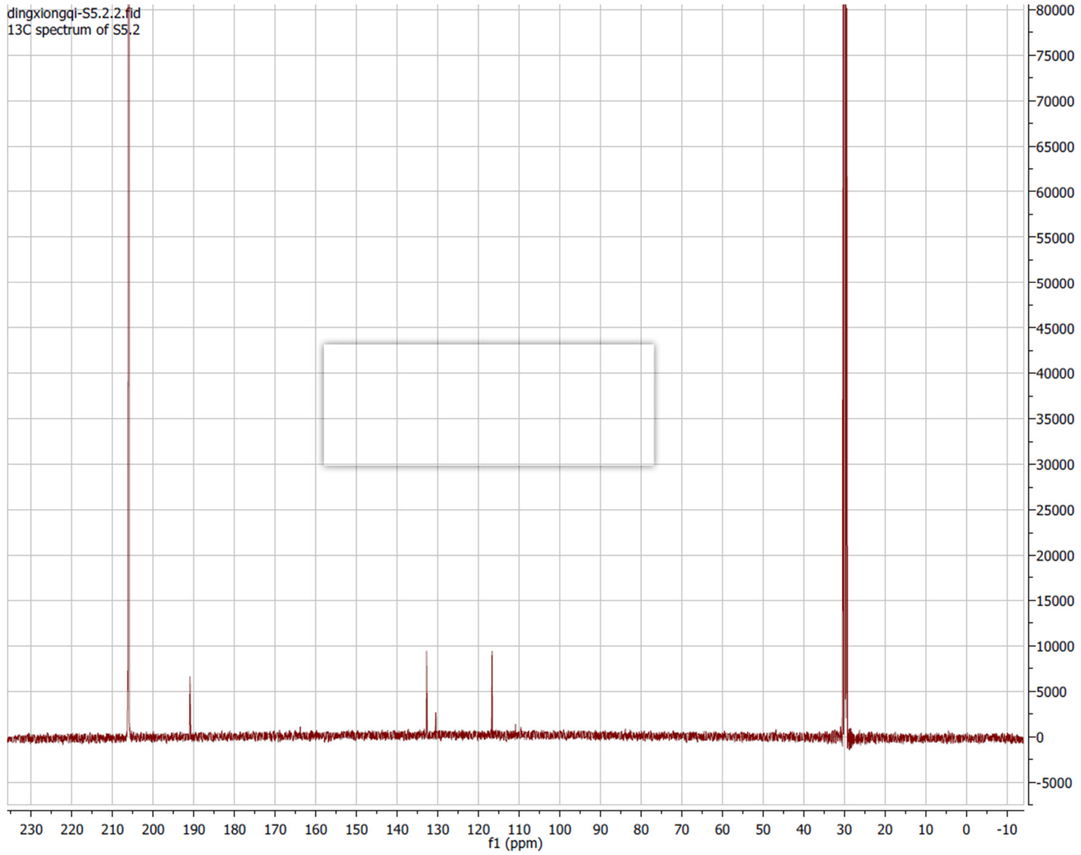
dingxiongqi-S5.2.2.fid
13C spectrum of S5.2
f1 (ppm)

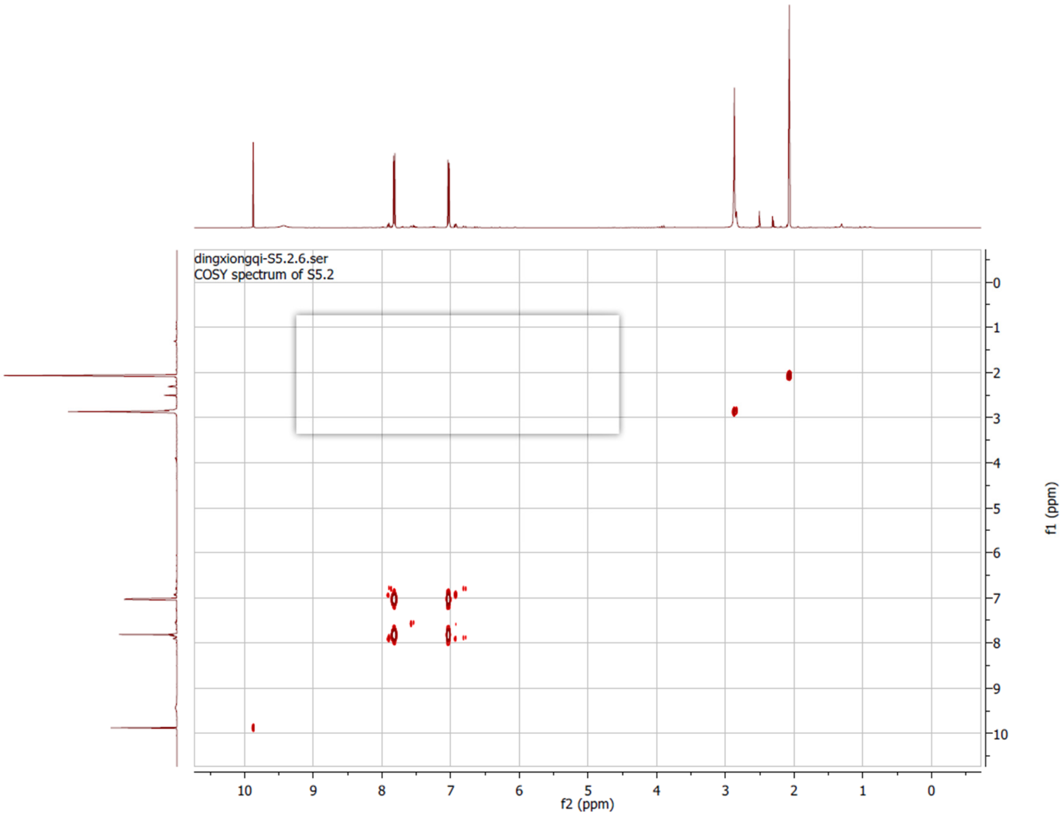
dingxiongqi-S5.2.6.ser
COSY spectrum of S5.2
f2 (ppm)
f1 (ppm)

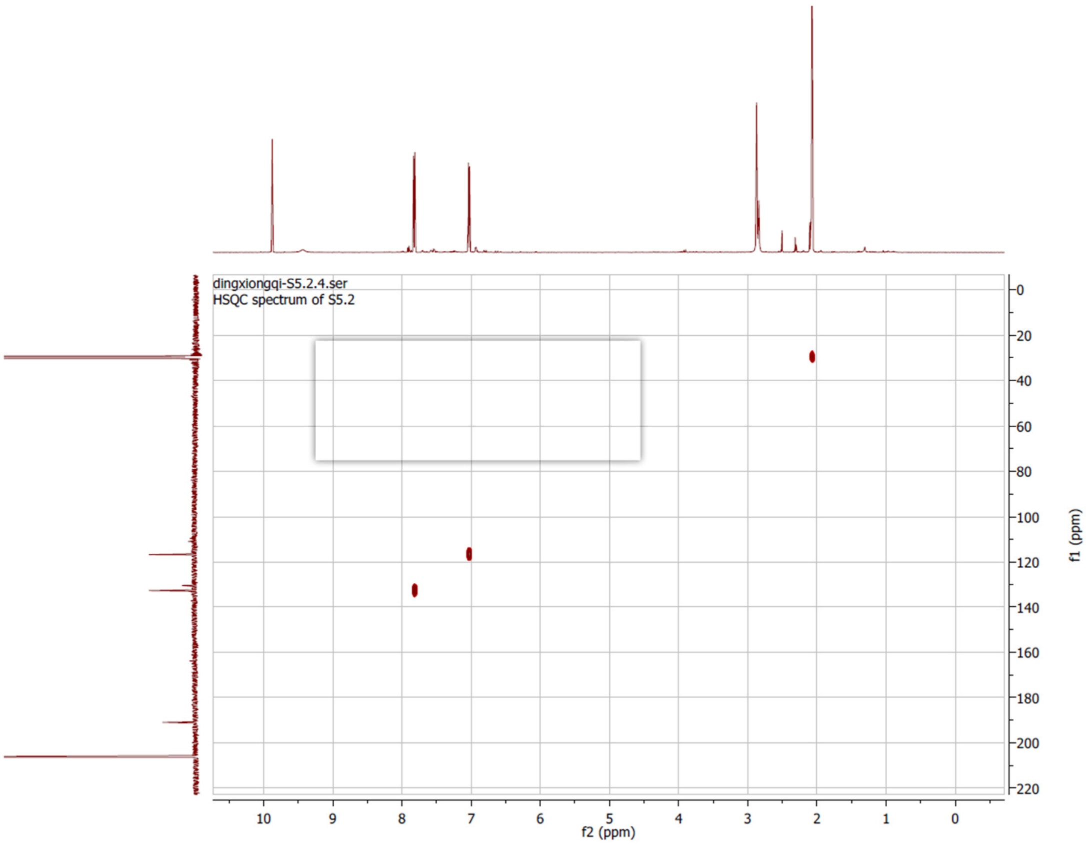
dingxiongqi-S5.2.4.ser
HSQC spectrum of S5.2
f2 (ppm)
f1 (ppm)

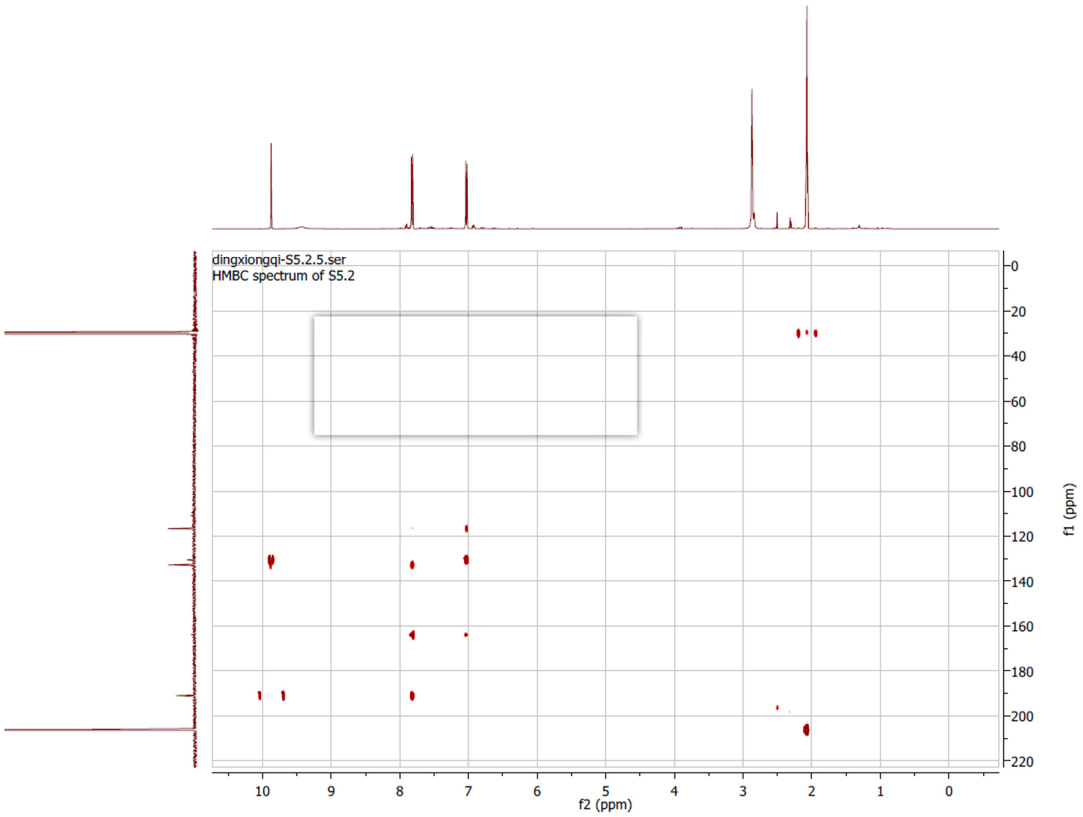

Figure S2: NMR 4-hydroxy-benzaldehyde.

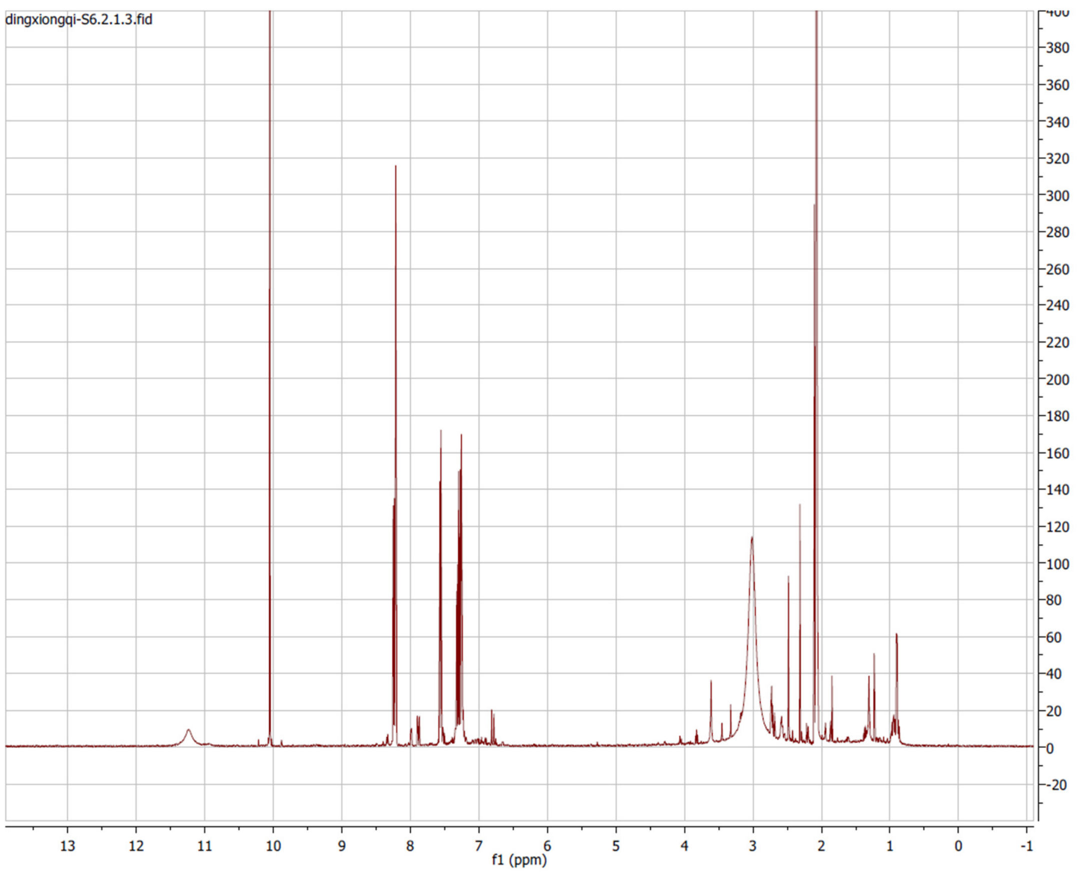
dingxiongqi-S6.2.1.3.fid
f1 (ppm)

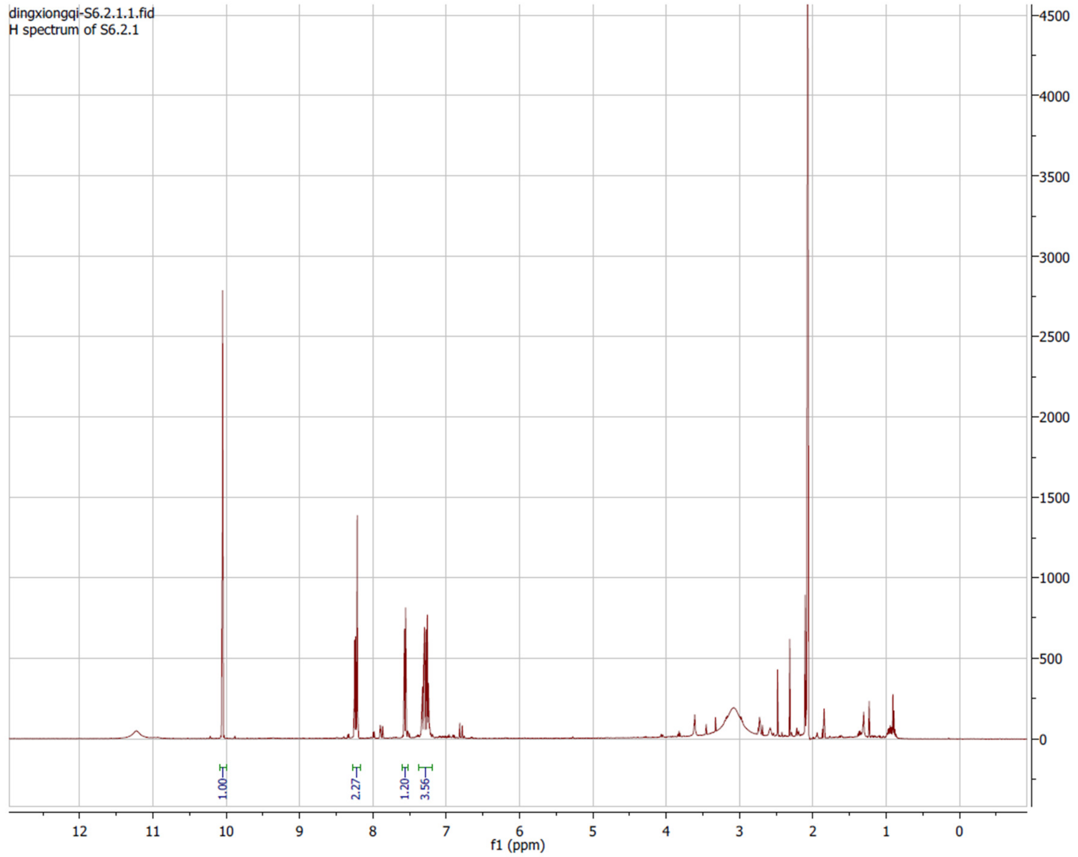
dingxiongqi-S6.2.1.1.fid
H spectrum of S6.2.1
1.00
2.27
1.20
3.56
f1 (ppm)

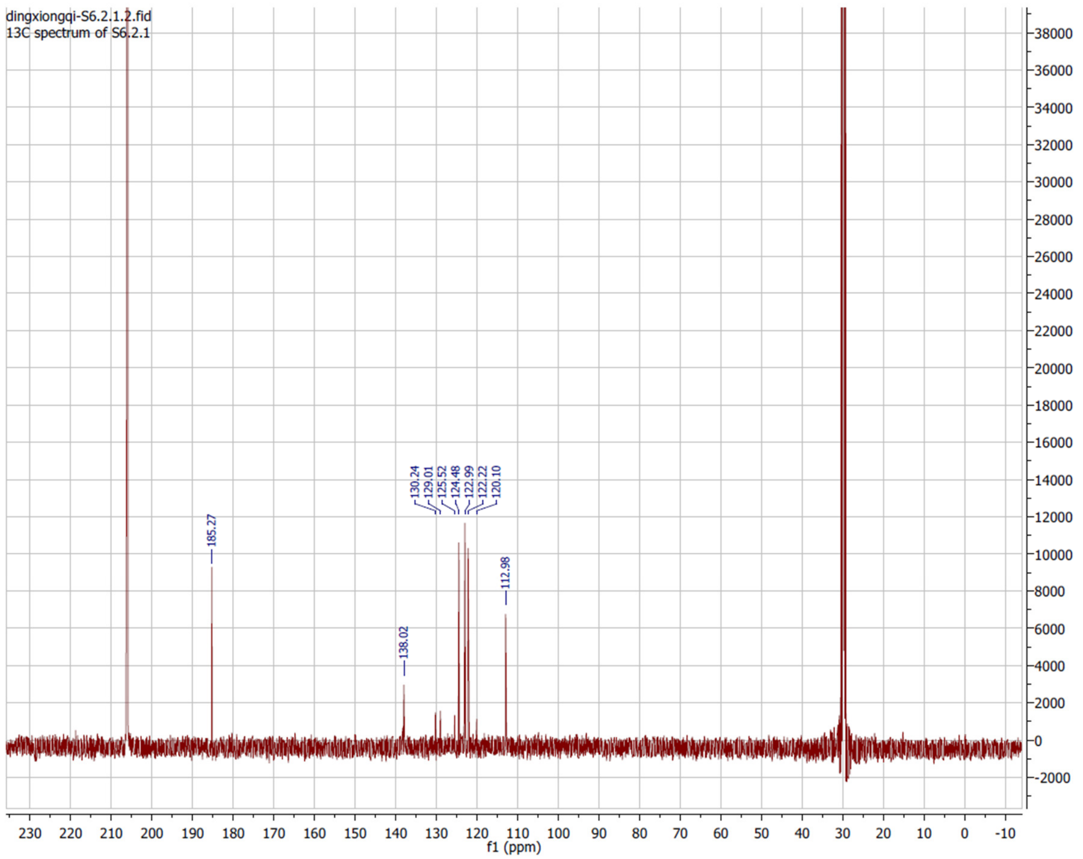
dingxiongqi-S6.2.1.2.fid
13C spectrum of S6.2.1
185.27
138.02
130.24
129.01
125.52
124.48
122.99
122.22
120.10
112.98
f1 (ppm)

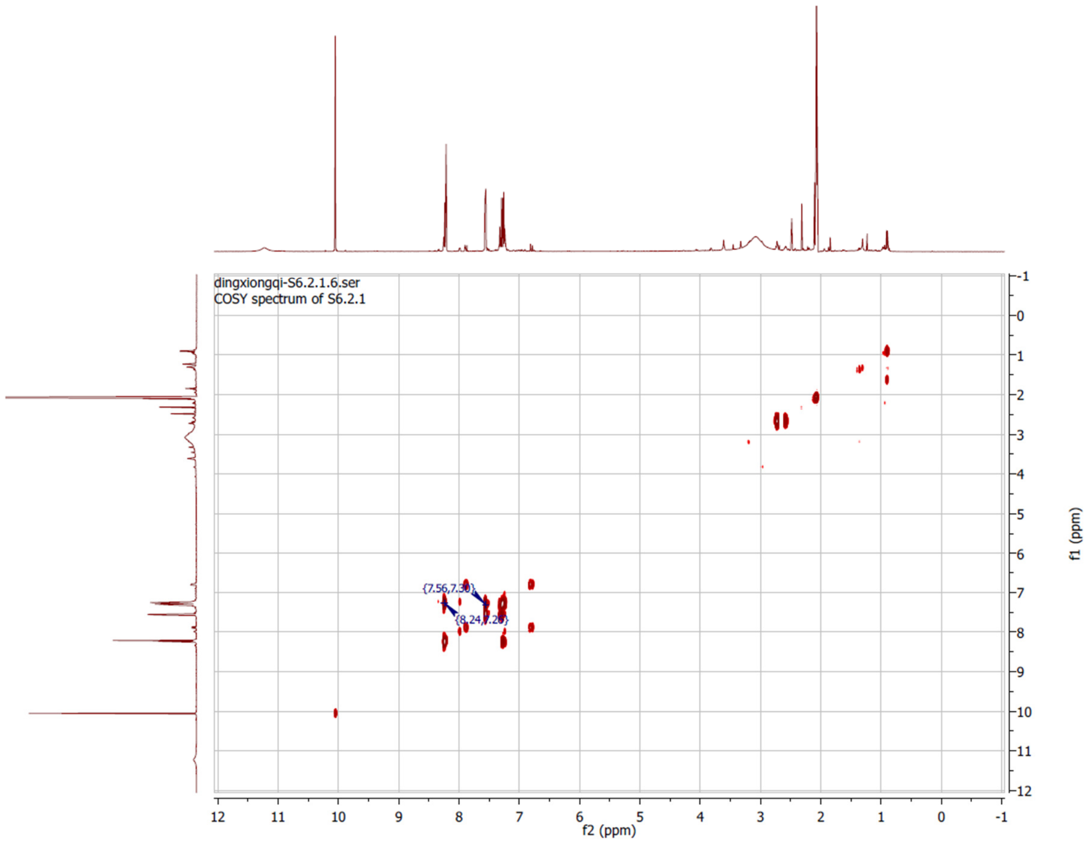
dingxiongqi-S6.2.1.6.ser
COSY spectrum of S6.2.1
{7.56,7.30}
{8.24,7.20}
f2 (ppm)
f1 (ppm)

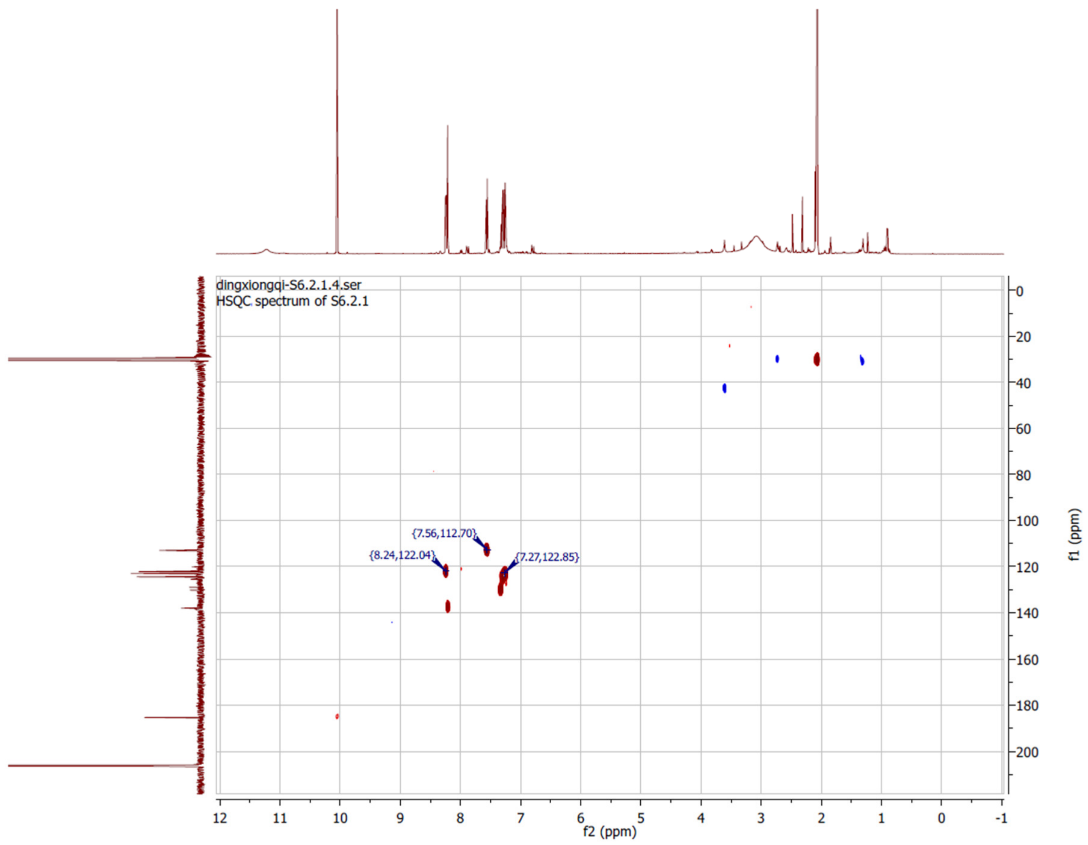
dingxiongqi-S6.2.1.4.ser
HSQC spectrum of S6.2.1
{7.56,112.70}
{8.24,122.04}
{7.27,122.85}
f2 (ppm)
f1 (ppm)

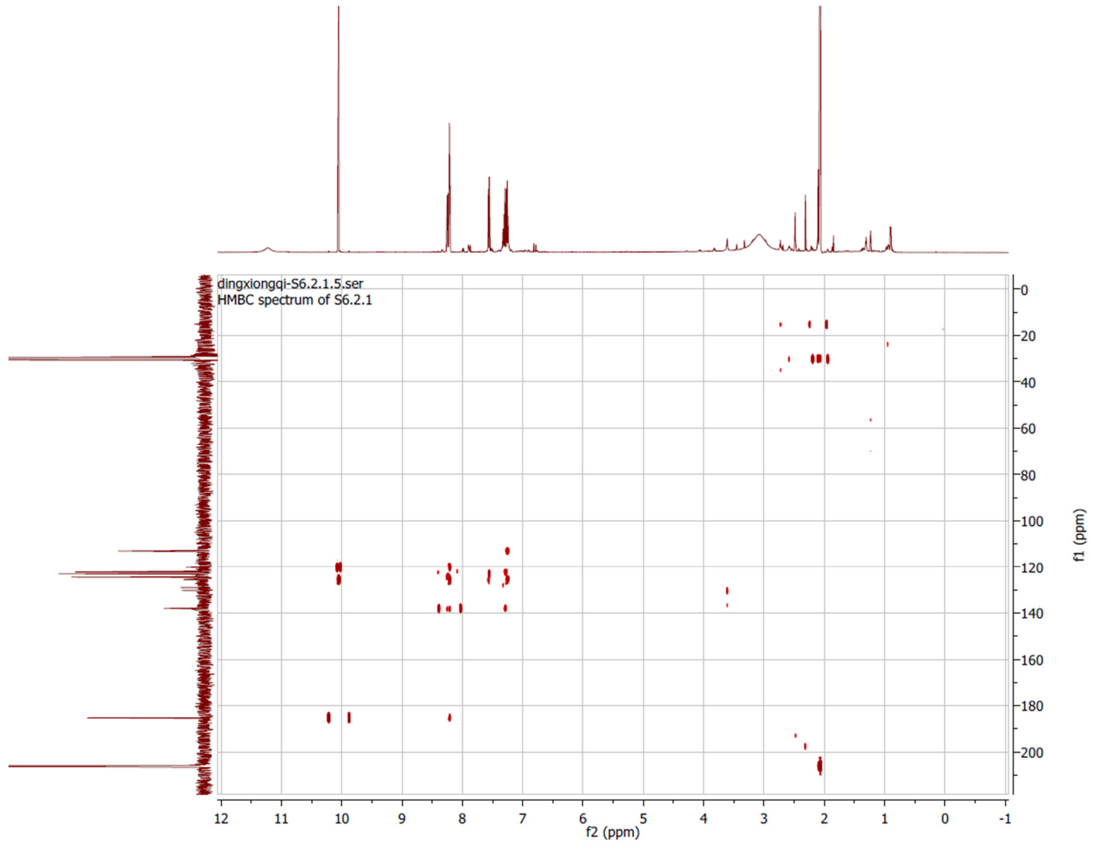
dingxiongqi-S6.2.1.5.ser
HMBC spectrum of S6.2.1
f2 (ppm)
f1 (ppm)

Figure S3: NMR 1H-indole-3-carboxaldehyde.
